# Strategies for increasing the use of tranexamic acid in patients undergoing major surgery*

**DOI:** 10.1002/anr3.12335

**Published:** 2024-11-28

**Authors:** L. Murphy, S. R. Warnakulasuriya

**Affiliations:** ^1^ Department of Anaesthesia Royal Free London NHS Foundation Trust London UK; ^2^ Department of Anaesthesia and Perioperative Medicine University College London Hospitals NHS Foundation Trust London UK; ^3^ NIHR Blood and Transplant Research Unit in Data Driven Transfusion Practice Oxford UK

**Keywords:** blood loss, surgical, blood transfusion, checklist, tranexamic acid

## Abstract

Tranexamic acid reduces major bleeding events in patients undergoing major surgery without increasing thromboembolic events. In October 2022, the Joint Royal Colleges Tranexamic Acid in Surgery Implementation Group issued recommendations for consideration of tranexamic acid use in all patients having inpatient surgery. National and local audit data shows that a significant portion of eligible patients do not receive tranexamic acid. We designed and implemented a quality improvement project to increase the use of tranexamic acid in patients undergoing major surgery (surgery with the potential for estimated blood loss > 500 ml). Data were collected on baseline tranexamic acid use and stakeholder‐reported barriers to tranexamic acid use. This was used to design and implement a sequence of quality improvement interventions. We disseminated Joint Royal Colleges guidance and delivered education sessions to increase understanding of tranexamic acid use. The local World Health Organisation (WHO) surgical checklist was updated to prompt clinical staff to consider the use of tranexamic acid. At baseline tranexamic acid was used in 50 of 100 (50%) major surgical cases. In the third audit cycle, tranexamic acid use had improved to 65 of 96 (68%) cases, with a shift in practice noted on continuous monitoring data indicating sustained improvement. Key factors in successful implementation of this project included stakeholder engagement, widespread dissemination of education and guidance and change of the local WHO surgical checklist.

## Introduction

Tranexamic acid is a synthetic lysine analogue which binds to and inhibits plasminogen, preventing plasmin from breaking down fibrin, thereby acting to stabilise existing clots. Use of intra‐operative intravenous tranexamic acid is a central intervention in peri‐operative patient blood management (PBM) [1], which aims to optimise patients undergoing surgery who are at risk of requiring a blood transfusion. Use of tranexamic acid is supported by recent emerging evidence that showed a reduction in major bleeding events in patients undergoing major surgery [2]. Meta‐analysis has shown no increase in thromboembolic events [3]. It is estimated that the implementation of tranexamic acid administration may prevent 15,000 major surgical bleeds per year in the UK [4]. This is particularly pertinent in the context of recent blood shortages which have resulted in cancellation of elective operations [5].

Implementation of peri‐operative patient blood management as a package has been shown to improve patient outcomes [6]. Whilst routine use of tranexamic acid is an established practice in orthopaedic surgery [7], its use is more sporadic in other specialities [8]. In October 2022 the Joint Royal Colleges Tranexamic Acid in Surgery Implementation Group recommended that ‘*consideration of tranexamic acid use*’ be included in the safe surgery checklist for inpatient surgical cases [4].

National audit data from the 2021 National Comparative Audit of NICE Quality Standard QS 138 shows that a significant proportion (32%) of eligible patients do not receive tranexamic acid [9] and this was reflected in local audit data. Previous service improvement work on perioperative PBM [10] had developed a list of procedures at our institution with potential for blood loss over 500 ml and this was used to develop our audit standard. We aimed to explore local barriers to the use of tranexamic acid and introduce a quality improvement (QI) project to increase uptake of intravenous tranexamic acid use in patients undergoing surgery with potential for blood loss over 500 ml, implementing evidence‐based research [2] to improve patient outcomes and meet Joint Royal Colleges recommendations and NICE guidance on patient blood management [1, 4].

## Methods

This project was approved by the local audit and QI panel as a local service improvement project. The QI project was conducted from January 2023 to July 2024 using Model for Improvement methodology at a single tertiary National Health Service (NHS) hospital and is reported according to SQUIRE guidelines [11] (Supporting Information S1).

Data were collected on two different groups of patients: Group A – two‐week cycles of all adult non‐obstetric patients who had a procedure with potential blood loss > 500 ml as defined by a local list of procedures (Supporting Information S2); Group B – continuous data monitoring using EPIC Slicer Dicer (EPIC systems Corporation 2023) of all patients with procedures audited in the National Comparative Audit of NICE Quality Standard QS 138 [9]. Data was stored within NHS information technology resources and analysed using Microsoft Excel (Microsoft 365, Version 2302; Microsoft Corporation, Redmond, USA). Group A categorical data were analysed using a Chi‐Squared test, chosen a‐priori. For the primary outcome of ‘cases receiving tranexamic acid’, a p‐value < 0.05 was considered significant. For the secondary five measures in Group A, the Bonferroni comparison was used (p‐value divided by number of measures analysed), resulting in a p‐value of 0.01 being considered significant. Group B was analysed using a statistical process control chart and presented as a p‐chart, with recalculation of process limits at the point of implementation of the World Health Organisation (WHO) checklist change.

Three audit cycles were carried out of patients in group A: baseline (9 January 2023); after commencement of presentations and roadshows (5 June 2023); and after introduction of an updated WHO laminated surgical checklist (31 July 2023). Data were collected on surgical speciality, procedure, dose and timing of tranexamic acid (in relation to surgical skin incision), estimated blood loss and venous thromboembolism risk factors. Cycles 1 and 3 were compared statistically as this represented the implementation of the main interventions.

After an initial ‘Plan, Do, Study, Act’ (PDSA) cycle, continuous monitoring of intra‐operative intravenous tranexamic acid was established to enable ongoing monitoring and sustainment of the project; EPIC's slicer dicer facility was used for the National Comparative Audit of NICE Quality Standard QS 138 [9] eligible procedures, to be representative of wider practice at our institution (data extracted for 1 January 2023 to 31 July 2024). The balancing measure of cost was estimated using pharmacy ordering data (for 500 mg tranexamic acid ampoules for the main University College Hospital campus, excluding the obstetric site).

After initial data collection in January 2023, barriers to implementation were explored using stakeholder interviews in March 2023. Consultant surgeons and anaesthetists (n=13) were asked about their threshold and exceptions for giving tranexamic acid, whether they would routinely mention it at ‘sign‐in’ and whether they were in favour or opposed to introducing a question into the WHO surgical checklist. A multi‐intervention plan was developed consisting of education, dissemination of guidance and changes to checklists (implementation chart in Supporting Information S3), which included:

- Education
  ○ Presentations to anaesthetists
  ○ Electronic communications to anaesthetists and surgeons
  ○ Surgical sub‐speciality tranexamic acid roadshows
    ■ Educational presentation and engagement of surgeons in focus groups
- Dissemination of Joint Royal College guidance
  ○ NHSBT ‘Is your patient having major surgery’ posters [12]
  ○ Link to RCS webinar provided during tranexamic acid roadshows [13] and in electronic communications
- Changes to the WHO checklist
  ○ Laminated sheets
  ○ Electronic checklists

## Results

Stakeholder interviews showed that surgeons and anaesthetists were positive about the idea of using tranexamic acid in patients with potential blood loss > 500 ml. The most frequently mentioned concern was the presence of current or previous venous thromboembolism (n = 6 out of 13). Ten out of 13 participants had heard of the Joint Royal Colleges guidance but were unsure of the details; one had changed practice in response to it. All participants were in favour of adding a question about tranexamic acid to the WHO checklist. Views expressed in individual interviews were representative of focus group discussions during roadshows, where surgeons also expressed concerns regarding data on what constituted potential for blood loss > 500 ml, impact of tranexamic acid on brisk arterial bleeding and the potential for tranexamic acid to cause clotting.

Our teams were receptive to the checklist change and no ‘checklist fatigue’ was observed. However, observation did reveal that an electronic checklist was routinely used rather than the updated laminated version; therefore, the electronic version was updated as well.

**Figure 1:**
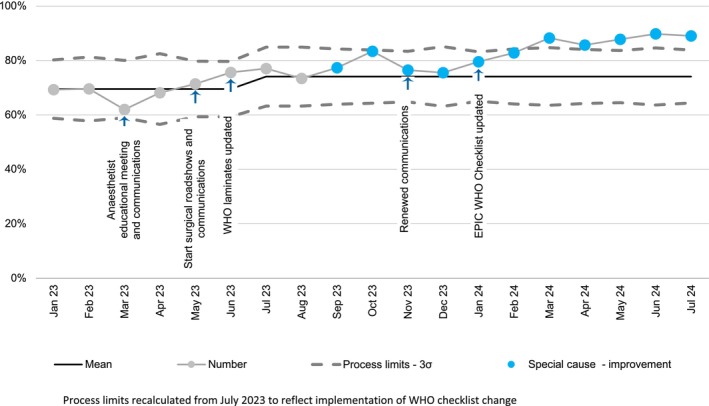
Statistical process control p‐chart showing proportion of tranexamic acid use over time in patients undergoing procedures at risk of ‘moderate blood loss’ according to the National Comparative Audit of Blood Transfusion.

Results of each data collection cycle are summarised in Table 1 and in Supporting Information S3. There was a significant increase in patients receiving tranexamic acid in cycle 3 (68%) compared to cycle 1 (50%). This improvement trend was greatest in non‐orthopaedic cases (Cycle 1 28%; cycle 3 55%; p = 0.002). Any difference in patients receiving tranexamic acid prior to skin incision was not statistically significant (Cycle 1 82%; Cycle 3 87%; p = 0.393). Review of patient notes in audit cycles 1 and 2 found no in‐hospital postoperative venous thromboembolism. The number of tranexamic acid ampoules ordered in 2022 was 13,445 and in 2023 it was 13,485. Continuous monitoring of tranexamic acid use in group B (n = 3177) showed a sustained increase in proportional use with special cause improvement (Fig. 1).

**Table 1:** Use of tranexamic acid in audit cycles 1 and 3 for all adult non‐obstetric patients who had a procedure with risk of estimated blood loss > 500 ml.

	Cycle 1	Cycle 3	p‐value
Cases with potential estimated blood loss > 500 ml; n	100	96	n/a
Cases receiving tranexamic acid; n (%)	50 (50)	65 (68)	**0.012**
Cases receiving tranexamic acid before surgical incision; n (% of all patients receiving tranexamic acid)	41 (82)	57 (87)	0.393
Orthopaedic cases receiving tranexamic acid; n (%)	31 (97)	32 (94)	0.591
Non‐orthopaedic cases; n	68	62	n/a
Non‐orthopaedic cases receiving tranexamic acid; n (%)	19 (28)	34 (55)	**0.002**
Non‐orthopaedic cases receiving tranexamic acid before surgical incision; n (% of non‐orthopaedic patients receiving tranexamic acid)	10 (53)	27 (79)	0.042
Cases receiving 2 doses of tranexamic acid; n (%)	1 (1)	5 (5)	0.087

Note: Bold values indicated statistical significance.

## Discussion

Our QI project shows the impact of strategies for engaging anaesthetists and surgeons in implementing guidance into local practice. Despite guidance being issued over multiple communication systems, there was a lack of local knowledge on the content of this guidance, demonstrating the need to increase local dissemination and discussion.

Tranexamic acid is an evidence‐based intervention shown to reduce surgical bleeding [2]. Our stakeholder interviews and focus groups revealed gaps in knowledge regarding its pharmacological action and recent evidence. Our targeted sub‐speciality focus groups successfully engaged surgeons and anaesthetists and allowed local champions to explain the evidence and address concerns.

Use of prophylactic tranexamic acid has been recommended for knee and hip replacement since 2020 [14] and baseline rates were high in our institution. Whilst it was not previously included in the published checklists, orthopaedic surgeons at our institution would routinely add a question to the time‐out process, which created precedent to introduce this question into the WHO checklist. We hypothesise that the introduction of this question into the checklist primarily affected non‐orthopaedic cases, which combined with the lower pre‐intervention use in this group explained the larger change in non‐orthopaedic patients.

Tranexamic acid is a relatively low‐cost intervention, costing approximately £1.50 to £3.10 per 1 g ampoule [15]. Our pharmacy data show that the number of vials ordered for the main hospital site increased slightly from 2022 to 2023, which was associated with increased peri‐operative use. As a balancing measure, this shows that our interventions did not create significant additional costs to our department's medicines budget.

Initial stakeholder educational events resulted in limited change in administration, in line with acknowledged issues with education related methodologies [16]. Changing our local WHO checklist was associated with a sustained increase in tranexamic acid administration. The observation that surgical teams were using an alternative electronic checklist version highlights the importance of observation in the clinical environment to ensure ‘work as imagined’ matches ‘work as done’ when implementing QI initiatives [17].

In terms of limitations, our main reported outcome is a process measure rather than a clinical outcome, therefore we cannot comment on clinical significance. Our sample size is not powered to detect differences in blood loss or venous thromboembolism. We also recognise that the knowledge of clinicians that data on tranexamic use were being collected may have contributed to the measured increase in its use.

Further work at our institution will look at promoting a two‐dose tranexamic acid administration protocol, with the second dose administered at the end of surgery, as recommended by the Joint Royal Colleges educational webinar [13]. We will also explore implementing a reminding function into the electronic health record, which may have a greater impact on administration [16].

Whilst there is published evidence on other national guidelines and PBM bundles as a whole, there is a paucity of reports on the implementation of tranexamic acid protocols. We hope our project may be of interest to institutions aiming to increase the uptake of these guidelines specifically with regards to tranexamic acid.

## Supporting information


**Data S1.** Standards for Quality Improvement Reporting Excellent (SQUIRE) 2.0 Guidance.

**Data S2.** UCLH procedures with risk of EBL > 500ml.

**Figure S1.** GANTT Chart.
**Table S1.** Use of tranexamic acid in audit cycles 1, 2 and 3 for all adult non‐obstetric patients who had a procedure with risk of estimated blood loss > 500 ml.
