# Supplementary material for: Strategies for increasing the use of tranexamic acid in patients undergoing major surgery*
Source: Anaesth Rep. 2024 Nov 28;12(2):e12335. doi: 10.1002/anr3.12335 (PMC11604225; doi:10.1002/anr3.12335)
Supplement: Supplementary file 2 — Data S2. UCLH procedures with risk of EBL > 500ml. [file ANR3-12-e12335-s002.pdf]

UCLH procedures with risk of EBL>500mls

Lower GI
Abdominoperineal (AP) resection
Anterior resection
Any colectomy/hemi-colectomy/proctectomy
Hartmann's procedure
Ileoanal anastomosis and creation of pouch
Ileo-caecal resection
Laparotomy (including exploratory)
Redo operations on ileum/colon
Resection of duodenal tumour
Resection of small bowel
Reversal of Hartmann's procedure
Transanal endoscopic microsurgery

Upper GI
Bypass/ Closure of bypass of oesophagus
Gastrectomy
Oesophagectomy (partial /total)
Open excision of lesion of oesophagus
Revision of anti-reflux procedures
Revision of gastro-jejunostomy
Splenectomy (partial/total)
Transabdominal anti-reflux operations
Transabdominal repair of diagphragmatic hernia
Transthoracic fundoplication
Transthoracic repair of diaphragmatic/hiatus hernia
Vagotomy and pyloroplasty
VATS excision lesion of oesophagus

Other Abdominal
Abdominal wall reconstruction
Adrenalectomy
Excision of retroperitoneal tumour/ lymph nodes
Restoration of intestinal continuity

Gynaecology
Block dissection of pelvic lymph nodes
Exenteration of pelvis (anterior/posterior/total)
Hysterectomy (laparoscopic/open/vaginal)
Radical vulvectomy
Reconstruction of vagina
Repair of rectovaginal fistula
Radical trachelectomy and removal of lymph nodes

Vascular
Endarterectomy of femoral artery
Open embolectomy of artery
Reconstruction for popliteal aneurysm

Orthopaedics
Complex pelvic osteotomies and fixation
Primary hip / knee replacement
Removal of total hip replacement
Revision of total hip/ knee replacement

Head and Neck
Craniofacial resection
Glossectomy (total)
Laryngectomy
Maxillectomy
Mediastinal thyroidectomy (with sternotomy)
Mediastinal parathyroidectomy (with sternotomy)
Pharyngectomy (partial / total)
Radical dissection of cervical lymph nodes
Radical tumour clearance with flap reconstruction
Reconstruction of jaw
Selective/radical dissection of cervical lymph nodes

Thoracics
Bullectomy
Correction of pectus deformity of chest wall
Decortication of pleura of the lung
Excision of chest wall tumour
Lung resection/ lung volume reduction
Mediastinal parathyroidectomy with sternotomy
Open excision of lesion of lung
Open resection of mediastinal tumour
Pleural biopsy (open)
Plication of paralysed diaphragm
Pneumonectomy
Posterior costoplasty
Pulmonary lobectomy
Repair of ruptured diaphragm
Sleeve resection of bronchus
Thoracoplasty
Thoracotomy
VATS

Urology
Appendiciovesicostomy / Mitrofanoff procedure
Bilateral replantation of ureter into bladder
Bilateral Ureterolysis
Construction of ileal conduit
Cystectomy
Enlargement of bladder
Enterocystoplasty
Excision of ureterocele
Ileal or colonic replacement of ureter
Open correction vesicoureteric reflux
Pelvic exenteration
Percutaneous nephrolithotomy
Prostatic cryotherapy
Radical prostatectomy
Repair of bladder exstrophy
Repair of vesicocolic fistula
Replantation of ureter into bowel
Retroperitoneal lymph node dissection
