# Supplementary material for: Strategies for increasing the use of tranexamic acid in patients undergoing major surgery*
Source: Anaesth Rep. 2024 Nov 28;12(2):e12335. doi: 10.1002/anr3.12335 (PMC11604225; doi:10.1002/anr3.12335)
Supplement: Supplementary file 3 — Figure S1. GANTT Chart. Table S1. Use of tranexamic acid in audit cycles 1, 2 and 3 for all adult non‐obstetric patients who had a procedure with risk of estimated blood loss > 500 ml. [file ANR3-12-e12335-s003.docx]

**Supporting Figure 1: GANTT Chart**


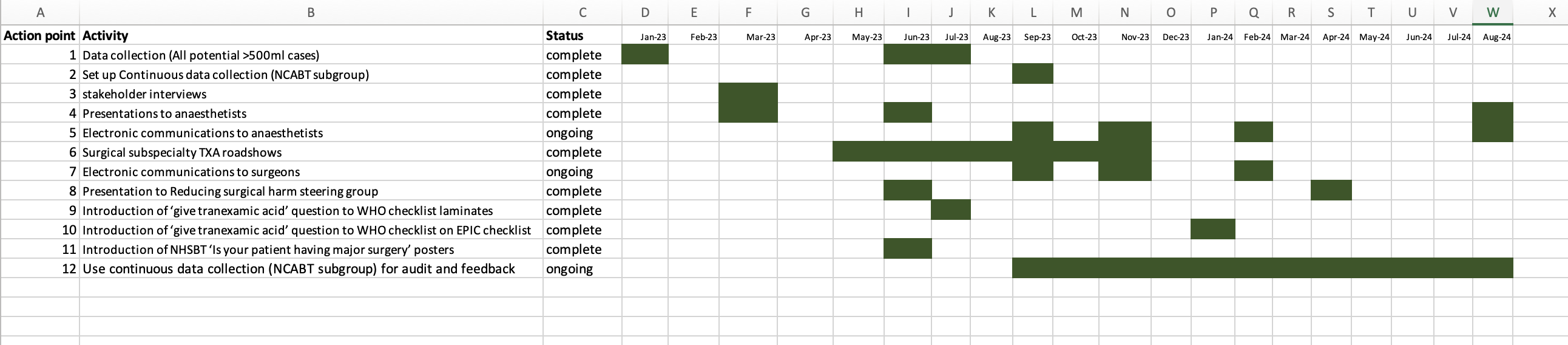


**Supporting Table 1. Use of tranexamic acid in audit cycles 1, 2, and 3 for all adult non-obstetric patients who had a procedure with risk of estimated blood loss >500ml**

| **Audit cycle (group a)** | **1** | **2** | **3** |
| --- | --- | --- | --- |
| **Cases with potential estimated blood loss >500ml; n** | 100 | 159 | 96 |
| **Cases receiving tranexamic acid; n (%)** | 50 (50) | 82 (51) | 65 (68) |
| **Cases receiving tranexamic acid before surgical incision; n (% of all patients receiving tranexamic acid)** | 41 (82) | 69 (84) | 57 (87) |
| **Orthopaedic cases receiving tranexamic acid; n (%)** | 31 (97) | 46 (81) | 32 (94) |
| **Number of non-orthopaedic cases** | 68 | 102 | 62 |
| **Non-orthopaedic cases receiving tranexamic acid; n (%)** | 19 (28) | 36 (35) | 34 (55) |
| **Non-orthopaedic cases receiving tranexamic acid before surgical incision; n (% of non-orthopaedic patients receiving tranexamic acid)** | 10 (53) | 25 (69) | 27 (79) |
| **Cases receiving 2 doses of tranexamic acid; n (%)** | 1 (1) | 8 (5) | 5(5) |
